# Supplementary material for: A Proteomic Atlas of Lineage and Cancer-Polarized Expression Modules in Myeloid Cells Modeling Immunosuppressive Tumor-Infiltrating Subsets
Source: J Pers Med. 2021 Jun 11;11(6):542. doi: 10.3390/jpm11060542 (PMC8230595; doi:10.3390/jpm11060542)
Supplement: Supplementary file 1 [file jpm-11-00542-s001.zip › jpm-1230337-supplementary.pdf]

Journal of Personalized Medicine

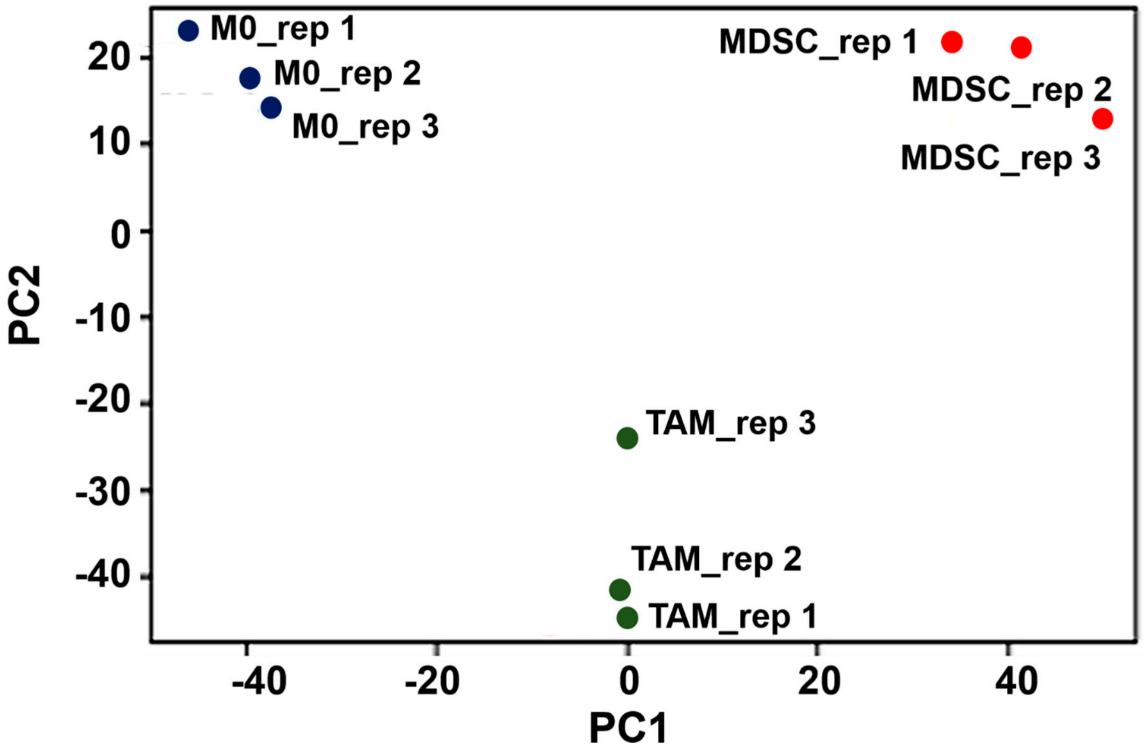

Supplementary Figure S1 . Phenotype of bone marrow-derived non-polarized macrophages, TAM-like macrophages and MDSC. Principal component analysis of myeloid cell types differentiated ex vivo. The graph plots a principal component analysis of each cell type (3 independent cultures).

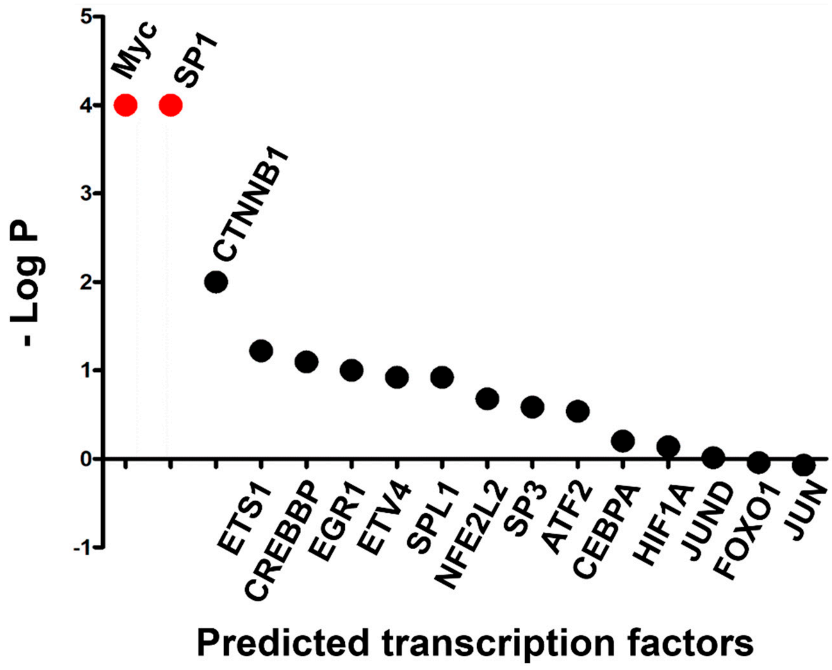

Supplementary Figure S2. Predicted transcription factors for the shared TAM and MDSC differential proteome. Dot plot graph representing the probability of each indicated transcription factor to be either activated in TAMs and MDSCs compared to non-

polarized M0 macrophages, using the TFACTs algorithm. Transcription factors with statistical significance of association to the differential proteomes according to probability (P<0.05) and false discovery rates (P<0.05) are highlighted in red.

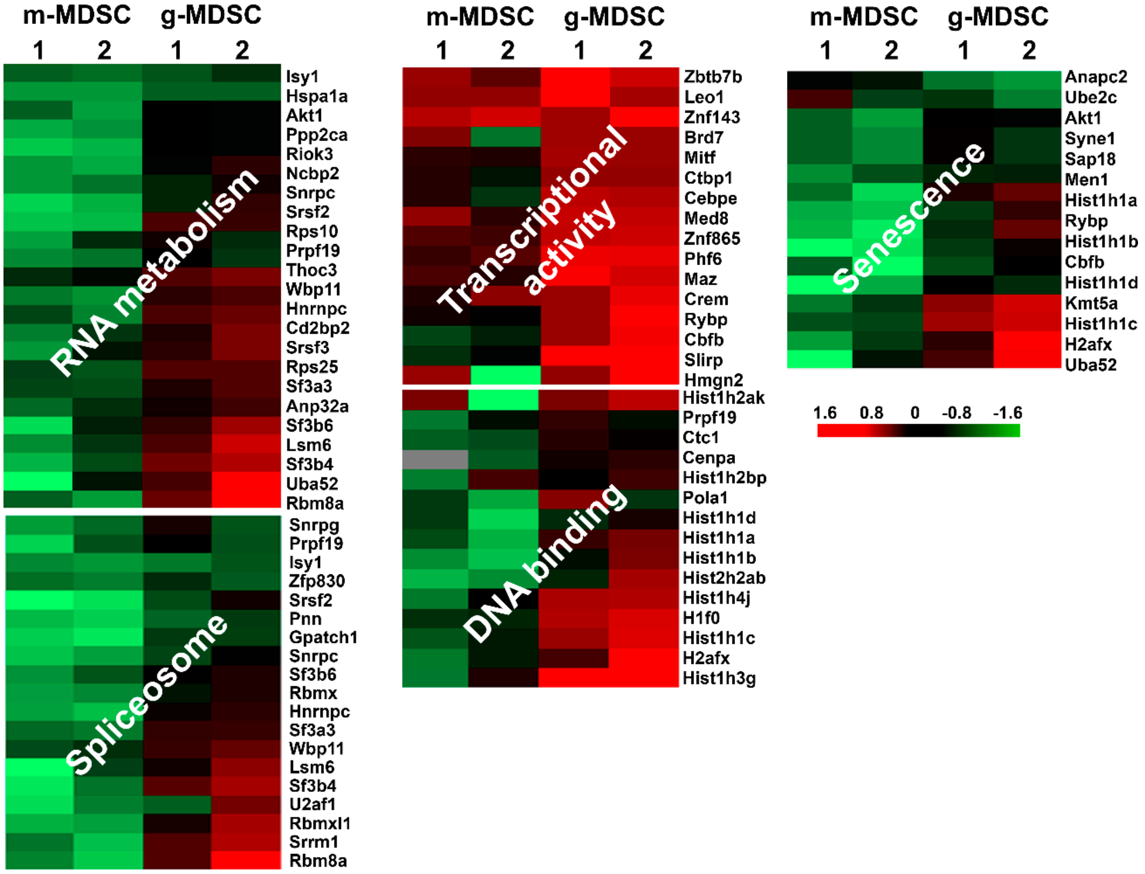

Supplementary Figure S3. Differential nuclear proteome between monocytic and granulocytic MDSCs. Heat maps of differential nuclear protein expression profiles (P<0.01) between monocytic and granulocytic MDSC cell cultures (duplicate independent biological replicates and purifications) as indicated. The legend (bottom right) indicates color-coded fold-change on log10. Red and green, up and down-regulated proteins, respectively.
